# Antigen Uptake in the Gut: An Underappreciated Piece to the Puzzle?

**DOI:** 10.1146/annurev-immunol-082523-090154

**Published:** 2025-04

**Authors:** Devesha H. Kulkarni, Rodney D. Newberry

**Affiliations:** 1 Department of Medicine, David Geffen School of Medicine, University of California, Los Angeles, California, USA; 2 John T. Milliken Department of Medicine, School of Medicine, Washington University, Saint Louis, Missouri, USA

**Keywords:** intestine, antigen, microfold cell, transepithelial dendrite, paracellular transport, goblet cell

## Abstract

The mammalian gut is a vast, diverse, and dynamic single-layer epithelial surface exposed to trillions of microbes, microbial products, and the diet. Underlying this epithelium lies the largest collection of immune cells in the body; these cells encounter luminal substances to generate antigen-specific immune responses characterized by tolerance at homeostasis and inflammation during enteric infections. How the outcomes of antigen-specific tolerance and inflammation are appropriately balanced is a central question in mucosal immunology. Furthermore, how substances large enough to generate antigen-specific responses cross the epithelium and encounter the immune system in homeostasis and during inflammation remains largely unexplored. Here we discuss the challenges presented to the gut immune system, the identified pathways by which luminal substances cross the epithelium, and insights suggesting that the pathways used by substances to cross the epithelium affect the ensuing immune response.

## INTRODUCTION

The mammalian gut represents a precarious environment for the immune system, as a single-layer epithelium separates trillions of microbes and microbial products from one of the largest collections of immune cells in the body. A central question in gut immunology is how immune cells, whose function is to protect the host from invading microbes, can be continually exposed to the wide array of foreign and potentially pathogenic substances from gut microbes and the diet, yet peacefully coexist in a mutually beneficial manner and remain poised to fight potential pathogens. This paradox has been a central subject of studies in gut immunology and remains incompletely explained.

Decades, if not centuries, of research have revealed how the immune system employs a wide variety of migratory and resident cell types and subsets to continually patrol the body to recognize and eliminate pathogens. These studies identified the elegant functions of individual cell types, the essentially limitless antigen receptor repertoire allowing the immune system to respond to almost any novel or previously seen pathogen, and the wide array of mediators that can coordinate immune responses between these cell types. In essence, these seminal observations explained how the immune system functions as a weapon to attack and eliminate invaders. More recent studies have provided insights into how this weapon is controlled to prevent untoward responses to self, resulting in tissue damage and autoimmunity. While these paradigms elegantly explain how the immune system recognizes and responds to invaders while avoiding damaging responses to self in sterile tissues—such as the lymph nodes and spleen, where all microbes might be viewed as foreign—such paradigms fail to elucidate how the immune system distinguishes friend from foe in the gut, where the immune system is exposed to identical products and antigens from pathogens and commensal microbes while simultaneously being exposed to antigens from the diet. Further complicating this paradox is that the gut immune system must be poised to mount inflammatory responses to pathogens, such that an inability to override tolerogenic response would likewise be detrimental. In concert, these observations suggest the existence of higher levels of control to allow the gut immune system to make decisions between tolerogenic or inflammatory responses that are not solely dependent upon recognition of foreign products or antigens.

Here we discuss a relatively underappreciated process, antigen uptake in the gut, which may hold keys to how the gut immune system balances these opposing and vital tasks and distinguishes friend from foe.

## REGIONAL VARIATION AND ORGANIZATION OF THE GUT

**Figure 1:**
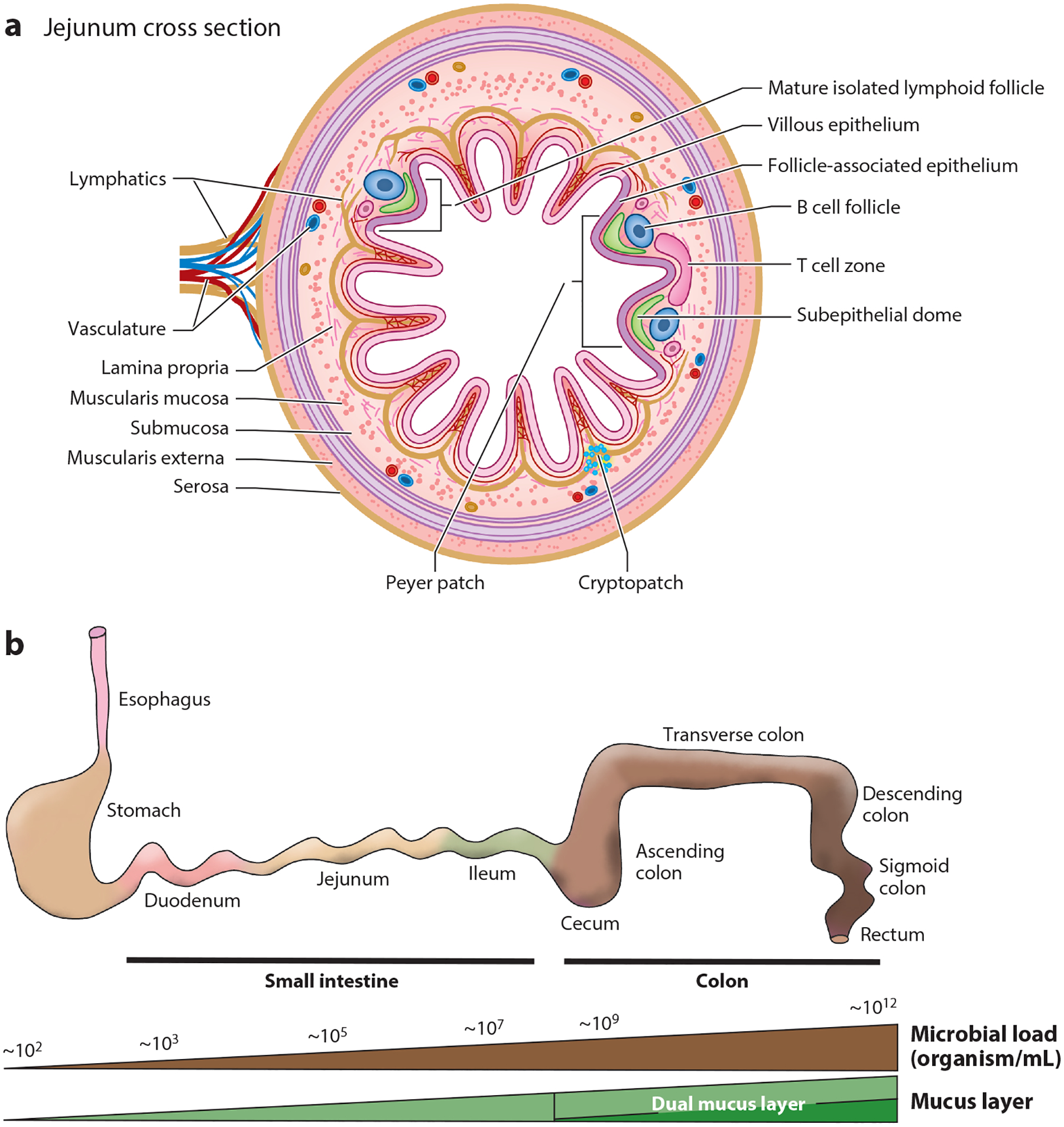
Regional variation in the gut environment. The gut displays a large regional variation in the luminal environment as well as in the niches/compartments containing immune cells. (*a*) Immune cells are located throughout the gastrointestinal tract and reside in organized lymphoid structures within the mucosa—cryptopatches, isolated lymphoid follicles, and Peyer patches (colonic patches in the colon)—and diffuse, less organized compartments such as the intraepithelial compartment, the lamina propria, and the muscle layers. (*b*) The luminal environment ranges from a low microbial load in the esophagus and stomach to an increasing density of microbes in the distal colon. Likewise, the mucus layer, which forms a physical barrier protecting the epithelium, becomes increasingly thicker from the proximal to distal gastrointestinal tract and becomes a two-layer barrier in the colon. Each of these aspects, as well as specialized functions of the regional sections of the gut, plays a role in how luminal substances are encountered to generate antigen-specific immune responses.

Adding complexity to the task of maintaining immunological homeostasis, the gut has a wide variation in the macro- and microenvironments in which the immune system functions (1–3) (overview in Figure 1). Such variation suggests that the immune system requires multiple approaches to ensure homeostasis. Indeed, studies of immune responses in the lymph nodes draining distinct segments of the gastrointestinal tract indicate that, at homeostasis, the character of the immune response varies not only by organ (e.g., small intestine versus colon) but also by region within an organ (4, 5).

The primary function of the gut is nutrient digestion and absorption, the specifics of which are beyond the scope of this article but are reviewed here (6). To accomplish these tasks, the gut has evolved region-specific functions. The stomach performs some of the first steps in digestion, including mixing and breaking down large food particles to be passed to the small intestine. The stomach has an acidic environment, which, in addition to aiding with digestion, may limit the number of microbes in the stomach (to ~10^2^/mL concentration) that are acquired from newly ingested food (3, 7). While not incapable of absorbing nutrients, the stomach absorbs nutrients in a limited capacity. Accordingly, the epithelial barrier in the stomach is viewed as less permeable and the underlying cellular immune components less dense than other portions of the gut at homeostasis.

The small intestine performs the majority of nutrient absorption and displays wide regional variation in the luminal environment as well as in function (2, 6). While the small intestine is traditionally divided into (from proximal to distal) the duodenum, jejunum, and ileum, recent studies have suggested a finer division of the small intestine based upon gene expression patterns and environment (1, 7, 8). The proximal small intestine has a less acidic environment, a higher concentration of digestive enzymes and bile acids, and a lower density of microbes (~10^3^–10^5^/mL concentration) than the rest of the gut (3, 7). With respect to nutrient absorption, macronutrients (protein, carbohydrates, fats) are largely, and very efficiently, absorbed throughout the small intestine; however, regional specializations for micronutrient absorption exist. The proximal small intestine is specialized for the absorption of minerals such as iron and calcium, and the distal small intestine is specialized for the absorption of bile acids, which play a critical role in the absorption of fat-soluble vitamins such as vitamins A, D, E, and K (9–12). In addition, the distal small intestine has log-fold increases in the density of microbes in the lumen (~10^7^/mL) (3, 7), adding further complexity to how the immune system integrates these cues.

In contrast to the small intestine, the colon largely does not absorb nutrients but primarily absorbs water (6). However, like the small intestine, the colon displays regional variations. The gut microbiota becomes more anerobic and much denser in the transition from the ileum to the cecum and reaches its maximum density in the distal colon (~10^12^/mL concentration) (3, 7). In concert with this change in microbial density and quality, the single layer of permeable mucus that lines the small intestine becomes a dual stratified layer in the colon. In the proximal colon, the inner mucus layer is less permeable than the small intestine, and in the distal colon the inner mucus layer becomes largely impermeable (13, 14), presumably to more effectively shield the epithelium from the increasingly dense microbial load. The luminal contents, aside from microbes, also change in the colon, with the content largely consisting of nondigestible fiber, which can be a substrate for gut microbes to produce metabolites such as short-chain fatty acids, which have an array of immunomodulatory capacities (15). Thus, given the regional differences within the gut, including the quantity and quality of microbes and the immunomodulatory cues to which the immune system is exposed, it is not surprising that multiple approaches that vary throughout the gastrointestinal tract may be required for the immune system to successfully handle threats and maintain homeostasis.

The gastrointestinal tract contains almost all the cellular components of the immune system and is enriched in some cellular subsets not commonly found in other sites in the body. The small intestine and colon are structurally similar, containing (from lumen inward) a mucosa consisting of a single-layer epithelium, the lamina propria and a thin muscle layer underlying the lamina propria, a submucosa containing stromal elements (lymphatics, vasculature, and a nerve plexus), a muscularis externa primarily containing two muscle layers and a nerve plexus, and a serosa consisting of a layer of connective tissue covering intraperitoneal portions of the gut. The intraperitoneal portions of the gastrointestinal tract are supported by a mesentery containing lymphatics and vasculature supporting the gut.

Each segment of the gut has a relatively distinct lymphatic drainage. Accordingly, those within the peritoneum drain into mesenteric lymph nodes (MLNs), while sections of the gastrointestinal tract that are located retroperitoneally (in the proximal small intestine and distal colon) are drained by lymph nodes located outside of the peritoneum (4, 7, 16). Note that all lymph nodes draining the small intestine and colon are often (incorrectly) referred to as MLNs. Organized lymphoid structures reside within the mucosa of the small intestine and colon. These can be divided into (*a*) secondary lymphoid structures—Peyer patches in the small intestine and cecal patches in the colon, which like lymph nodes develop in utero—and (*b*) solitary intestinal lymphoid tissues (SILT), which develop after birth along pathways paralleling secondary lymphoid tissue development (17). SILT are a spectrum of clusters of immune cells ranging from cryptopatches, containing lymphoid tissue inducer cells and dendritic cells (DCs), to isolated lymphoid follicles (ILFs), containing an organized B cell zone, T cells, DCs, and lymphoid tissue inducer cells (17, 18). Overlying the Peyer patches, cecal patches, and fully developed mature ILFs is a specialized follicle-associated epithelium (FAE) that is enriched in microfold cells (M cells; discussed in more detail below), which are specialized antigen-transporting cells (19). The organized lymphoid structures are scattered throughout the intestine with an increased density in the distal small intestine (Peyer patches and ILFs) and the cecum (cecal patch) (17, 20). Despite being distributed throughout the intestine, the organized lymphoid structure and overlying FAE containing M cells represent a vast minority (approximately <0.01%) of the surface area of the intestine.

The majority (approximately >99.9%) of the gastrointestinal tract is covered by a single-layer, non-follicle-associated epithelium. Within this single-layer epithelium resides populations of immune cells, including conventional and nonconventional T lymphocytes and mononuclear phagocytes (20, 21). Underneath this single layer of epithelial cells, within the lamina propria of the gut, lies one of the largest collections of immune cells in the body (20). Early studies of gut immunology largely focused upon Peyer patches and to a lesser extent cecal patches, as these structures were easily identified, cellular populations were easy to isolate, and the ability of M cells within the FAE to transport antigens to the underlying immune cells to generate antigen-specific immunity was readily apparent (22). Only more recently (in the last several decades) have studies more intensely focused upon the expansive cellular populations residing in the lamina propria, in part due to technical difficulties in isolating these cells when compared with isolating cells from Peyer patches and lymph nodes (18, 23). While it was appreciated that the lamina propria was a site with extensively active immune responses to luminal substances, how this occurs, and the implications of sampling luminal antigens by the lamina propria immune cells, remained less understood. Beneath the lamina propria, within the muscle layer, resides a sparser population of immune cells, largely macrophages, or microglia, that facilitate the development and function of the enteric nerves within the myenteric plexus (24, 25). The function of this immune cell population is only recently being revealed, and how it interacts with the lamina propria immune cells and luminal substances remains obscure. In addition, the esophageal mucosa and gastric mucosa contain sparser immune cell populations (26, 27). Most studies of these compartments evaluated these cellular populations during pathological processes. How these cellular populations interact with luminal substances and coordinate responses with other cellular populations in the gastrointestinal tract is largely unexplored.

## EVIDENCE FOR ANTIGEN UPTAKE IN THE GUT

When one is considering how luminal substances traverse the gut epithelium, it becomes important to remain cognizant of the ultimate outcomes and accordingly the implications defining this process. Antigen uptake, as implied by the name, suggests that acquisition of luminal substances will result in antigen-specific immune responses. Setting aside B1 B cell responses, which can be relatively less specific and T cell independent, this would suggest that substances crossing the epithelium are (*a*) the size of whole-protein molecules (many kilodaltons) or (following digestion in the lumen) (*b*) at least the size of peptides able to bind MHC molecules to initiate T cell–dependent immune responses (~2 kDa).

While the idea that substances large enough to induce antigen-specific immune responses cross the gut epithelium seems a given today, this has not always been the case. In traditional studies of macromolecular uptake, largely focusing on the primary function of the gut, the pattern of nutrient absorption indicates that macronutrients are digested and then absorbed as small molecules in the small intestine. Carbohydrates are broken down into monosaccharides; proteins are digested into free amino acids, dipeptides, and tripeptides; and lipids are digested into monoglycerides and free fatty acids, for uptake into enterocytes by specific transporters or free diffusion (6, 28). Digestion and absorption of small molecules account for the vast majority of dietary substances crossing the epithelium, and perhaps, given the error in measuring uptake in early studies, small molecules could be considered the entirety of ingested substances crossing the gut epithelium. However, more than half a century ago, substances up to the micrometer range could be found in the blood following oral administration in animals and humans (29). While not widely accepted as a physiological event at the time, this phenomenon was termed persorption because it was felt to be an abnormality involving particles crossing the epithelium through nonphysiological pathways such as barrier breach and muscular contractions (30, 31). Furthermore, this process was felt to contribute to disease (32–34), and the possibility that this process could be an integral part of mucosal immunity was largely not entertained.

A central, yet incompletely understood, phenomenon of gut immunology is oral tolerance (35, 36), a process whereby ingested substances induce a state of immune nonresponsiveness upon rechallenge. The process of oral tolerance, or tolerance to innocuous substances from the diet and commensal microbes, is thought to be fundamental to maintaining homeostasis in the precarious environment of the gut (37, 38). Accordingly, how this occurs and is maintained has been the focus of numerous studies. Studies revealed that low doses of oral antigen induced a state of active suppression in which the immune system actively suppressed responses to the ingested substance whereas high doses of oral antigen induced a state of anergy in which the immune system lost the capacity to respond to the orally administered antigen (39–41). Anergy was attributed to orally ingested antigens reaching the thymus to induce deletion of T cells with specificity for that antigen, similar to the process of negative selection to self-antigens, while active suppression remained an enigma (42, 43). Later, the discovery of T regulatory cells (Tregs) (44) helped to clarify what was occurring in low-dose oral tolerance: Luminal antigen was captured by lamina propria DCs that migrated to the draining lymph node to induce Tregs with antigen specificity to the luminal antigen, which suppressed immune responses to the antigen (35, 45). While these observations supported the idea that macromolecules could cross the intestine and have purposeful/physiological roles, where and how these substances crossed the epithelium to be encountered by the immune system remained unclear.

The discovery of M cells (19) (discussed below) overlying the FAE of Peyer patches, and later identified in the epithelium overlying mature ILFs, provided a potential answer to how luminal substances might be transcytosed across the intestine in a purposeful manner to generate antigen-specific tolerogenic responses. While antigen-specific tolerogenic responses to luminal substances could be induced in Peyer patches, multiple approaches led to the conclusion that Peyer patches were not required for tolerance to orally administered antigens; rather, oral tolerance was dependent upon the lymph nodes draining the intestine (MLNs) (46), which received antigen from DCs migrating from the lamina propria (45). Thus, while the uptake of macromolecules from the gut lumen to induce antigen-specific immune responses has been well established, how such uptake occurs in the villous epithelium, or non-follicle-bearing epithelium, which covers the vast majority of the surface of the gut, has not been well understood.

## PATHWAYS OF ANTIGEN UPTAKE

**Figure 2:**
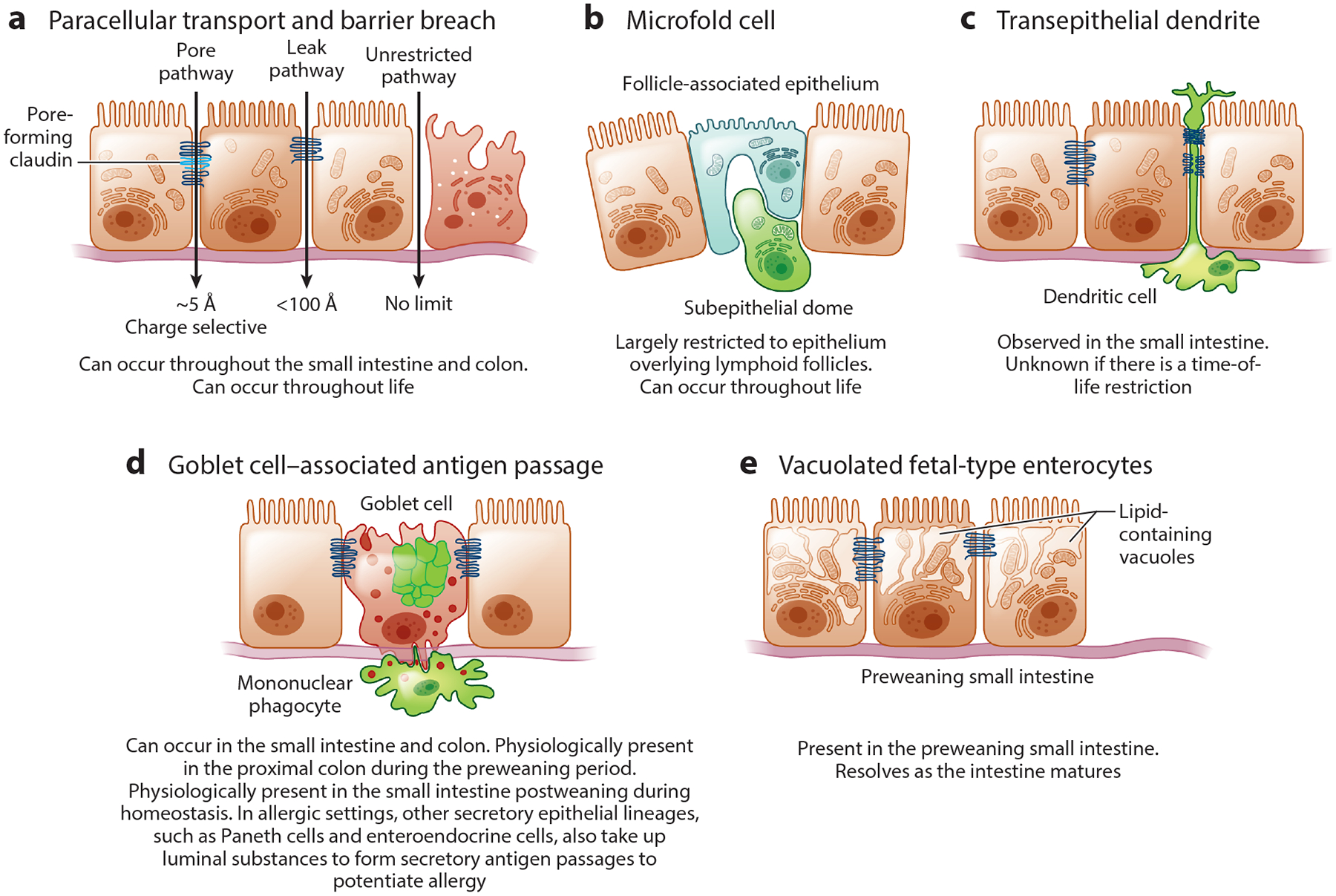
Pathways for luminal substances to cross the intestinal epithelium. Multiple pathways have been identified as potential mechanisms to support antigen-specific immune responses to luminal substances. (*a*) The paracellular transport pathway and barrier breach. The pore pathway, induced by the insertion of pore-forming claudins into the tight junctions, allows for passage of very small molecules and is charge selective. The paracellular leak pathway, induced by changes in tight junctions, allows for passage of larger molecules in a non-charge-selective manner. The unrestricted pathway, induced by barrier breach due to loss of epithelial cells, has no limitations with respect to size or charge. (*b*) Microfold cells (M cells) are largely restricted to the follicle-associated epithelium overlying the Peyer patches, colonic patches, and mature isolated lymphoid follicles. M cells transcytose luminal substances and deliver them to immune cells in the underlying subepithelial dome. (*c*) The extension of transepithelial dendrites by dendritic cells in the lamina propria allows for direct capture of luminal substances. Dendritic cells can express tight junction proteins, allowing these cells to traverse the tight junctions without disrupting the barrier. *(d)* Goblet cell–associated antigen passages form by a fluid-phase endocytic process that atypically delivers luminal cargo into the transcytotic pathway, where it is captured by lamina propria mononuclear phagocytes. During allergic conditions, goblet cells as well as other secretory epithelial lineages (Paneth cells and enteroendocrine cells) form secretory antigen passages to take up luminal substances to potentiate allergic responses. (*e*) Vacuolated fetal-type enterocytes occur in the small intestine of preweaning mammals. The vacuoles are largely lipid containing and are believed to form due to the uptake of milk. The vacuolated enterocytes resolve as the intestine matures. Panel *a* adapted from Reference 49 with permission from Springer Nature.

Having established that large molecules of a size capable of inducing adaptive immune responses cross the gut epithelium and that the outcome of this process can be beneficial and accordingly purposeful, we pose the question, How does this process occur? Multiple pathways by which large molecules cross the gut epithelium have been described (Figure 2 and Table 1). However, the means by which these pathways are controlled and the situations in which each pathway is most relevant remain largely unexplored.

### Paracellular Pore and Paracellular Leak Pathways

Our survival is dependent upon the ability of the gut to extract and absorb water, solutes, and macro- and micronutrients. These processes necessitate development of a concentration gradient across the epithelium and accordingly the presence of a selectively permeable transepithelial barrier. Desmosomes, adherens junctions, and tight junctions (basal to apical) join epithelial cells to form this selective barrier. Aside from micronutrient and macronutrient absorption, paracellular transport is likely the most studied process allowing passage of luminal substances across the epithelium. Paracellular transport is a controlled process whereby substances traverse the epithelium by passing through tight junctions between adjacent intestinal epithelial cells (47). This process occurs by two pathways (Figure 2a).

The first, the pore pathway, involves pore-forming molecules, such as claudins, located near the apical surface of epithelial cells. Pore-forming claudins insert themselves into the tight junctional complex between epithelial cells and allow small molecules (solutes) to pass between epithelial cells (48). Pores have a high capacity for flux that is both charge and size selective, with the size of the pores estimated to be ~5 Å (49), theoretically correlating to a spherical molecule of ~5 Da. This is well below the estimated size of molecules needed to initiate adaptive immune responses and suggests that, while the pore pathway of paracellular transport is controlled and could occur anywhere there is a single-layer simple epithelium in the gastrointestinal tract, it is not capable of delivering antigens to induce antigen-specific immunity.

The second pathway is the paracellular leak pathway, which, in contrast to the pore pathway, is not charge selective and has a low capacity. Like the pore pathway, the leak pathway is regulated at the level of tight junctional complexes, which can be modulated by increased production of tight junction proteins, removal/degradation of tight proteins, and modulation of the cytoskeleton. Activity of the paracellular leak pathway is most often assessed by detection of gav-aged 4-kDa fluorescent dextran in the serum (49), which is the range of the size of peptides that could stimulate adaptive immune responses. This pathway has been estimated to have the capacity to accommodate molecules the size of albumin (65 kDa) (47), but this is likely a rare situation, as loss of large molecules such as albumin into the gut lumen would be detrimental. Thus, the paracellular leak pathway is regulated; is potentially active throughout the gastrointestinal tract, which is lined by a simple single-layer epithelium; and has the potential to deliver substances to initiate adaptive immunity but may be limited for this purpose by low bidirectional flux of molecules.

### Barrier Breach

As noted above, the gastrointestinal tract is lined with a semipermeable epithelium that is largely impermeable to macromolecules in the homeostatic state. Disruption of this barrier occurs due to loss of epithelial integrity, which can allow the passage of substances of potentially unlimited size to the underlying immune system (Figure 2a). Barrier disruption allowing macromolecules to freely cross the epithelium is considered a pathological process that occurs both due to mechanical or chemical injury and during uncontrolled inflammation, such as that which occurs during inflammatory bowel disease and some enteric infections (47, 50).

The absorptive enterocytes/colonocytes composing a simple single-layer epithelium that lines the small intestine and colon is continually replaced every ~5 days by the generation of new cells from stem cells at the base of the crypt. An exception to epithelial discontinuity as a pathological process is anoikis (51), a process that occurs during programmed cell death and shedding of intestinal epithelial cells at the tip of villi or the crypt surface. During physiological epithelial shedding, a discontinuity the size of the shed epithelial cells forms, yet this gap in the epithelium is not permeable to macromolecules (52–54). Importantly, these gaps do not contain a nucleus and are not goblet cells (53), and they are hence not related to the goblet cell–associated antigen passages (GAPs) discussed below. Tight junction proteins from neighboring epithelial cells rapidly redistribute to close this gap in a matter of minutes, thus maintaining barrier integrity (55, 56). In total, these observations suggest that barrier disruption, while potentially contributing to disease pathogenesis through antigen delivery, is unlikely to be a mechanism delivering luminal antigens to the immune system in the homeostatic state.

### Microfold Cells

One of the most interesting mechanisms described for luminal surveillance is the capture and transcytosis of microparticles by specialized epithelial M cells (Figure 2b). As described above, M cells are found in the epithelium overlying Peyer patches. The primary function of M cells is thought to be antigen delivery to bona fide DCs for antigen presentation (57); hence, they are believed to be neutral agents without direct influence on the type of immune response generated.

M cell–mediated transcytosis, which includes antigen capture, transport of luminal particles across the mucosal barrier, and delivery to the submucosal tissue, requires unique properties (58). Like neighboring enterocytes, M cells are derived from stem cells in the intestinal crypt and have a lifespan of ~5 days (59). In contrast to nutrient absorption, the capture and transport of macromolecules from the apical to basolateral side of the epithelium in the homeostatic state is less common and largely restricted to M cells, which are a rare epithelial cell type, and goblet cells, which were only recently recognized to have this capacity (60).

Multiple studies have sought to identify M cell–specific capture receptors that might explain the process of microbial capture and transcytosis. However, it remains unclear how the receptor specificity for pathogen particles would be able to distinguish between commensal versus pathogenic microbes, in the same way that the mucosal immune response in general cannot make this distinction. Over the years, M cell–specific gene expression has yielded a number of capture receptor genes such as the gene encoding glycoprotein 2 (gp2), which is thought to bind the FimH protein on *Salmonella* (61). Mice lacking gp2 have reduced *Salmonella* dissemination to Peyer patches and thereby a reduction of antibody production against *Salmonella* (61). The lipid A domain of the lipopolysaccharide moiety found on gram-negative bacteria binds Annexin A5, a protein expressed on the apical side of M cells (62). *Salmonella* flagellin was defined as a key factor in promoting M cell differentiation through its capacity to trigger secretion of C-C motif chemokine ligand 20 (CCL20) by epithelial cells, a chemokine-inducing recruitment of B cells and DCs involved in the conversion of epithelial cells into M cells. Along the same line, the *Salmonella*-secreted SopB protein increases host invasion by converting epithelial cells in the FAE into M cells (63).

### Transepithelial Dendrites

DCs are professional antigen-presenting cells (APCs) that play a critical role in initiating adaptive immunity against enteric pathogens and establishing tolerance to dietary and commensal antigens (64–66). Highly specialized subsets of APCs are found in the gut, likely due to the high load of commensal antigens, the constant threat of potential pathogens, and the need to support tolerance in the homeostatic state. Over the years, studies have reported that APCs can send transepithelial dendrites (TEDs) from the lamina propria that penetrate through the tight junction and capture luminal antigens (65, 67) (Figure 2c). Extension of TEDs has often been associated with CX3CR1^+^ macrophages, where the Toll-like receptor (TLR) ligands or pathogenic bacteria enhance TED formation (68, 69). The extension of TEDs has been considered a favored route to support the induction and maintenance of tolerance during homeostasis, since the formation of TEDs does not compromise the epithelial barrier. However, recent discoveries have highlighted some limitations in the ubiquitous role of TEDs in antigen uptake, including the absence of TEDs in some mouse strains, which do not demonstrate defects in oral tolerance (70).

Moreover, which lamina propria DC subset dominates TED formation has been a topic of debate, as some studies demonstrated that CD11c-YFP-expressing lamina propria DCs formed TEDs predominantly in the proximal jejunum in response to TLR signaling, which differed from initial descriptions of TED formation by CX_3_CR1 cells (68, 69). In another study, researchers argued that CD103^+^ DCs are more adept than CX_3_CR1^+^ cells at coordinating adaptive immunity toward luminal antigens. Using two-photon microscopy and fluorescent reporter mice, these researchers showed that *Salmonella* challenge resulted in CD103^+^ DC migration from the lamina propria toward the gut epithelium and captured the pathogen (71). Furthermore, the stimuli inducing TED formation have varied between studies, with some studies identifying CX_3_CL1/Fractalkine expressed on epithelial cells in the ileum driving this process (69) and other studies suggesting a broader population of lamina propria DCs forming TEDs driven by chemokines, such as CCL20 (68). TED formation may not be restricted to lamina propria DCs. Lysozyme-expressing Peyer patch DCs have been described to extend dendrites around M cells to capture bacteria or particles (72). While most studies of enteric infection have readily identified TEDs, the frequency of TED formation in the steady state has varied between studies. Some have observed that the formation of TEDs is rare in the steady state but that they are induced by the removal of luminal contents and the mucus layer (73), a practice common to most approaches studying the formation of TEDs. Lactic acid and pyruvic acid were found to promote TED formation by CX_3_CR1^+^ cells (74), thus raising the possibility that epithelial cell stress induced by removing the luminal contents and mucus layer may promote TED formation in the homeostatic intestine. Thus, while the formation of TEDs by lamina propria DCs is a well-accepted occurrence, the cell types forming TEDs, the regional location of TED formation in the intestine, the various settings inducing TED formation, and the downstream events remain to be clarified.

### Goblet Cell–Associated Antigen Passages

While evaluating the presence and frequency of TEDs using two-photon in vivo imaging, researchers made the surprising observation that high-molecular-weight (10–70-kDa) model luminal antigens traversed the small intestine epithelium in columns containing a nucleus (60). These structures were identified as goblet cells (60), specialized secretory cells in the intestinal epithelium whose role has been traditionally thought of as producing and secreting mucins to maintain the mucus barrier. Accordingly, these structures were termed goblet cell–associated antigen passages, or GAPs (60) (Figure 2d). By in vivo imaging, GAPs were observed to deliver luminally administered fluorescent ovalbumin to CD11c^YFP+^ lamina propria DCs, and deletion of goblet cells resulted in the failure of lamina propria DCs to acquire antigen and stimulate antigen-specific T cells ex vivo (60). GAPs were found in the small intestine of all mouse strains and humans in the steady state and were therefore viewed as a part of normal physiology (60). By in vivo two-photon imaging, GAPs were observed to be absent from the colon, but this picture was later refined as techniques to image GAPs evolved to allow imaging of the distal colon (rectum, sigmoid colon, and descending colon), whereby GAPs were observed to be present, albeit at low density (5).

During in vivo imaging, GAPs were observed to form over a period of minutes and to regress/empty slowly over 1–2 h, suggesting that GAPs are dynamic and potentially regulated (60). Subsequent work revealed that GAPs were induced by acetylcholine (ACh) acting on the muscarinic ACh receptor 4 (mAChR4) and were inhibited by activation of the epidermal growth factor receptor (EGFR) either directly by EGFR ligands or indirectly via transactivation through Myd88 signaling via TLRs or the IL-1 receptor (75, 76). These observations had significant implications in that GAPs were highly regulated, and demonstrated that the absence of GAPs in the proximal colon (cecum, ascending colon, transverse colon, and proximal descending colon) in the steady state in adults was due to dense colonic gut microbiota sensed via TLRs and signaling via Myd88 to transactivate the EGFR and inhibit goblet cell responsiveness via mAChR4 to form a GAP (75). GAPs in the distal colon were found to be induced by ACh acting on mAChR3, which is not inhibited by EGFR activation, consequently allowing GAPs to form in the steady state (77).

The initial description of GAPs suggested that they preferentially delivered luminal substances to CD103^+^ lamina propria DCs (60), an observation that was later refined to include all APC subsets in the lamina propria except for B cells (73). The characteristics and size of the luminal substances delivered via GAPs are broad ranging, from small inert molecules to microbial products and live bacteria (5, 78, 79). In addition, goblet cells and GAPs serve as a portal for entry of enteric pathogens (76, 78, 80–82).

The cellular biology and structural basis of GAP formation have only recently been elucidated. GAPs form through an atypical fluid-phase endocytic process that uncharacteristically delivers luminal substances to the transcytotic pathway (77), a pathway in enterocytes that is restricted to receptor-mediated endocytosis. The endocytic vesicles of GAP formation were observed to have the same membrane protein markers as the membranes enclosing the mucin granules in the goblet cell theca (77). In addition to the transcytotic pathway, some of the luminal substances and membranes were trafficked to lysosomes and the trans-Golgi network, presumably for incorporation into newly formed mucin granules (77). This recycling of membranes is thought to allow a highly secretory cell, such as a goblet cell, to efficiently maintain a high-secretory state without the energy requirements of generating entirely new membranes for secretion. The same stimulus, ACh, induced GAP formation, via activation of mAChR4 (or mAChR3 in the distal colon), and mucin secretion, via mAChR1, but used two different intracellular signaling pathways (77). This allows the goblet cell to form a GAP in the absence of mucin secretion, to secrete mucin in the absence of GAP formation, or to do both in parallel. Thus, this scenario explains how goblet cells can maintain the protective mucus barrier and not form GAPs in situations in which the luminal environment may be viewed as hostile and unfavorable for immune sampling such as in the proximal colon. Goblet cells near the tip of the villus were more likely to form GAPs in response to ACh, whereas those in the crypts were more likely to secrete mucin in response to ACh (77), suggesting that GAP formation may be a component of maturation as goblet cells advance from the crypt to villus tip. In other work, endocytic capacity, like that in GAPs, was found to extend to other intestinal epithelial cells in the secretory lineage (Paneth cells and enteroendocrine cells) in the allergic setting (83). These structures were termed secretory antigen passages (SAPs) and were induced in an ACh-independent manner by IL-13 (83). SAPs deliver ingested food allergens across the epithelium to induce mast cell degranulation and anaphylaxis in food allergy–conditioned mice.

### Vacuolated Fetal-Type Enterocytes

Decades ago, a time-limited specialized form of uptake macromolecular substances from the gut lumen was discovered to occur in early life in animals and humans through the formation of vacuolated fetal-type enterocytes (84, 85). Vacuolated fetal-type enterocytes are intestinal epithelial cells with an atypical apical canalicular system, resulting in giant-sized vacuoles present in utero and shortly after birth in the small intestine and regressing in a proximal-to-distal fashion during the first few weeks of life as the epithelium matures in a process termed closure (85–88) (Figure 2e). Vacuolated fetal-type enterocytes take up luminal immunoglobulins (from breast milk) and other macromolecules (85, 87). Colostrum induces uptake of luminal substances by vacuolated fetal-type enterocytes and induces their disappearance with maturation of the intestinal epithelium (87). Studies have not explored the ability of vacuolated fetal-type enterocytes to deliver luminal substances to the immune system to generate antigen-specific immunity, but given the relative immaturity of the immune system at the time when vacuolated fetal-type enterocytes are present, they are unlikely to play this role.

## COULD ANTIGEN UPTAKE BE PART OF THE PROCESS DISTINGUISHING FRIEND FROM FOE?

Having established that luminal substances cross the epithelium via many different mechanisms and that there are antigen-specific responses to luminal substances encountered by the immune system, we pose the following questions: Does the pathway taken to encounter the immune system impact outcomes on the ensuing immune response, and is this pathway part of the larger schema distinguishing friends from foes in this potentially hostile environment? Unfortunately, this question is largely unapproached, and many illustrations of immune responses in the gut depict antigen uptake with simple arrows crossing the epithelium, suggesting that antigen uptake and ensuing immune responses result from constitutive and unregulated antigen delivery, with no specific inputs by how the antigen was acquired. While data to directly address this question are limited, some insight into the role of antigen uptake pathways in immune outcomes can be gained from examining when and the contexts in which these pathways are active.

M cells are restricted largely to the FAE overlying Peyer patches, cecal patches, and mature ILFs. These structures are present in the steady and pathological states, although mature ILFs increase in number following stress (89, 90). Indeed, Peyer patches and mature ILFs are sites that are relatively uniquely adept at generating antigen-specific IgA responses (91, 92), a hallmark of immune responses for both maintaining homeostasis and fending off pathogens in the gut. Furthermore, Peyer patches are sites for the induction of tolerance to luminal antigens, yet as noted above, this is redundant, as gut-draining lymph nodes, not Peyer patches, are essential to generate oral tolerance. Whether transcytosis of antigens by M cells can be modulated is not clear; however, pathogens can induce the generation of M cells in the FAE (93, 94). Such generation, to the benefit of the pathogen, may provide portals for entry into the host and, from the host side, further suggests that immune responses downstream of M cell antigen delivery may be protective.

The immune response downstream of antigen capture by APCs extending TEDs into the lumen has been inferred to be both tolerogenic and inflammatory, but data supporting these outcomes are incomplete. The presence and frequency of TED extension in the homeostatic state are controversial, which may be related to the generation of TED-inducing metabolites by the gut microbiota. Furthermore, the extension of TEDs has not been observed in the distal colon, where tolerance to luminal substances can be induced (5, 73, 95). In support of the extension of TEDs promoting a protective/inflammatory response, TED extension is augmented by enteric infection, and genetic models show that mice lacking TED extension are more susceptible to enteric infection. However, an alternative explanation for TED extension is provided by studies indicating that the extension of TEDs may be an initial step in the transmigration of APCs into the gut lumen during enteric infection, which has an important role in limiting the luminal pathogen load.

Barrier breach has been associated with inflammatory responses, in part due to the fact that barrier breach most often occurs during injury or infection or in the setting of intestinal inflammatory disease. As such, it is inferred, although largely uninvestigated, that barrier breach promotes inflammatory responses to luminal antigens. The outcomes of immune responses downstream of paracellular transport by leak are also largely unexplored. While this pathway is active in the steady state, during which tolerogenic responses predominate, stress induces inflammatory cytokines and activates the leak pathway (96), facilitating the delivery of macromolecules of a size capable of inducing adaptive immunity, suggesting that this pathway is induced by, if not facilitating, antigen-specific inflammatory responses.

The best evidence that antigen delivery pathways influence the phenotype of the immune response comes from studies of GAPs. GAPs are present in the non-follicle-bearing epithelium (60), indicating that they deliver antigens to impact immune responses in the draining lymph nodes, which can contain antigen-naive T cells and potentially, although not yet investigated, deliver antigen to differentiated T cells in the lamina propria. GAPs are a major antigen uptake and delivery pathway supporting tolerance to luminal antigens in the small intestine and distal colon in the steady state (73). In addition, GAPs are present in the proximal colon during a defined preweaning period, where they are necessary to induce long-lived peripherally derived Tregs (pTregs) specific for some commensal microbes and dietary antigens (97, 98). These early-life pTregs have the long-term effects of supporting a balanced immune system and controlling allergic responses (98, 99). However, as tolerance is the dominant tone of the intestinal immune system in the steady state, these observations may simply be the result of GAPs delivering luminal antigens to a tolerogenic environment as opposed to GAPs having a role in shaping immune responses. Supporting the latter possibility, GAPs are closely regulated and are inhibited when the luminal environment would not be favorable for sampling luminal antigens, such as during enteric infection and in the setting of the abundant colonic gut microbiota (75, 76). Overriding GAP inhibition in these settings does not induce tolerance but rather induces inflammatory responses to innocuous luminal antigens (73), translocation of resident commensal microbes in the colon (100), and increased pathogen dissemination (76). Further supporting that the role of GAPs goes beyond delivering luminal antigens, inhibition of GAPs in the small intestine, where they would be present in the steady state, has the surprising effects of rapid reductions in the Treg population in the lamina propria, expansions in the Th17 population in the lamina propria, and loss of imprinting lamina propria APC populations with tolerogenic properties (73). While the basis for these events is unclear, they are too rapid to be explained by loss of antigenic stimulation on Tregs and de novo generation of Th17 cells. Mechanistically, these events might be explained by observations that, when APCs sample from GAPs, they acquire goblet cell proteins (60) and by observations that the goblet cell protein Mucin 2 can imprint DCs with tolerogenic properties and can induce antigen-specific Tregs (101). While it is understandable that loss of APC imprinting would impair generation of de novo pTregs, how loss of lamina propria APC imprinting contributes to the expansion of Th17 cells and the antigen specificity of the expanded Th17 cells remains to be explored.

## CONCLUSION

Clearly much remains to be explored and learned about the pathways of antigen uptake in the gut and their contributions to the ensuing immune response. Equally clear is that evidence suggests that this process should no longer be simplistically viewed as an indiscriminate, unregulated, route-agnostic, and constitutive process without implications for downstream events. In addition to the gaps in our understanding about the roles of the individual pathways of antigen uptake outlined above, future studies will also face the challenge of how these pathways work together in the varied landscape of the gut to ensure immune homeostasis in the precarious environment of the gut and how these processes may become altered to contribute to disease.

## Figures and Tables

**Table 1:** Pathways for luminal substances to cross the intestinal epithelium

Pathway(s)	Function(s) in antigen update	Location(s)	Known substances delivered	Reference(s)
Microfold cells	Immune surveillance	Peyer patches	Pathogenic bacterial adhesion, fungi	19, 58, 61, 63
Transepithelial dendrites	Immune surveillance, tolerance	Small intestine	Bacteria, food antigens	65, 67–69
Paracellular pores and leak	Small molecules and solutes	Small intestine and colon	Water, solutes, small molecules	47, 49
Goblet cell–associated antigen passages	Oral tolerance	Small intestine and distal colon (proximal colon in infancy)	Dietary antigens, commensal microbes, viruses, pathogenic bacteria	60, 73, 97
Secretory antigen passages	Food sensitization	Small intestine	Food allergens	83
Vacuolated fetal enterocytes	Found during infancy	Jejunum, duodenum	Immunoglobulins, milk components	84, 85, 87, 88
